# Links across disabilities: unveiling associations between functional domains

**DOI:** 10.1186/s12889-023-17523-5

**Published:** 2024-01-02

**Authors:** J. Dalal, S. Mitra, A. James, M. Rivas Velarde

**Affiliations:** 1 Geneva School of Health Science, University of Applied Sciences Geneva HES-SO, Geneva, Switzerland; 2 Department of Economics, Fordham University, Bronx, USA; 3 Department of Public Health, Erasmus MC, University Medical Center, Rotterdam, The Netherlands

**Keywords:** Disability, LMICs, Health-inequities, Disability-disaggregated data, Functional difficulties, Correlations/associations, Rights of disabled people

## Abstract

**Background:**

Persons with disabilities experience higher risks of mortality as well as poorer health as compared to the general population. The aim of this study is to estimate the correlations between functional difficulties across several domains in six countries.

**Methods:**

National census data with questions on disability from six countries (Mauritius, Morocco, Senegal, Myanmar, Vietnam, and Uruguay) was used in this study. We performed logistic regressions to assess the extent to which having a functional difficulty in one domain is correlated with having a functional difficulty in each of the other domains and report weighted odds ratios (ORs) overall and within age-groups (‘18–44’ years and ‘45+’ years). Models adjust for age, sex, and location (rural or urban). Sensitivity analyses around different choices of predictors and response variables were conducted.

**Findings:**

For all countries, reporting a functional difficulty in one domain was consistently and significantly positively correlated with reporting a functional difficulty in other domains (overall) and for each of the two age-groups considered - ‘18–44’ years and ‘45+’ years. All ORs were greater than one. Cognition, mobility, and hearing were the domains that were the most correlated ones with other domains. The highest pairwise correlations were for i/ hearing and cognition; ii/ mobility and cognition. Results were robust to changing the severity thresholds for functional difficulties. Across countries, Uruguay, the only high-income country among the six countries under study, had the lowest correlations between functional domains.

**Conclusions:**

There are consistent positive associations in the experience of functional difficulties in various domains in the six countries under study. Such correlations may reflect barriers to social services including healthcare services and resources (e.g. assistive devices) that may lead to an avoidable deterioration of functioning across domains. Further research is needed on the trajectories of functional difficulties and on structural barriers that people with functional difficulties may experience in their communities and in healthcare settings in particular. This is important as some functional difficulties may be preventable.

**Supplementary Information:**

The online version contains supplementary material available at 10.1186/s12889-023-17523-5.

## Introduction

Around 16% of the world population (approximately 1.3 billion people) live with disability, as of 2021 [1]. Disability is very diverse, and the results of the interaction between the individual’s health condition, such as blindness, with personal and environmental factors. Some persons are born with disabilities, others acquire disabilities due to illness, injury, or aging [1]. There has been increasing evidence over the years suggesting that persons with disabilities experience higher risks of mortality as well as poorer health compared to the general population [1, 2]. Compared to people without disabilities, disabled people are at a higher risk of developing secondary conditions (for instance, depression, anxiety etc.) and co-morbid conditions (high blood pressure, cardiovascular disease, and diabetes), having unintentional injuries, premature mortality, age-related conditions, health-risk behaviors (smoking, alcohol consumption, lack of physical activity etc.), and being exposed to violence (especially women; see [1, 3]). This can be partly attributed to an unequal access to healthcare services resulting in more unmet healthcare needs compared to the rest of the population [1, 4]. A 2022 study conducted in the context of Colombia, examining the existing healthcare-inequities, showed that deaf persons have significantly poorer health than the rest of the population and experience potentially preventable loss of function in relation to mobility, sight, cognition, communication, and self-caring [5]. For instance, persons with some intellectual disabilities die up to 20 years earlier than persons without disabilities [6]. Furthermore, hearing loss has been linked with cognitive decline, particularly in older adults [5].

There is limited data regarding health inequities experienced by persons with disabilities in Low- and Middle-Income Countries (LMICs). This is partially due to the challenge in defining, measuring, and collecting disability disaggregated data. The definition used terminology and even instruction to those collecting data heavily impact results. National instruments that use stigmatizing language tend to report lower rates of disability. With the view of standardizing disability measures suitable for census and national surveys, the United Nations Washington Group has developed and tested a set of questions focusing on six functional domains: seeing, hearing, walking, cognition, self-care, and communication. This set of questions is called the Washington Group Short Set of Questions (WG-SS). The WG-SS uses a four-level answer scale (no difficulty, some difficulty, a lot of difficulty, and unable to do). As the results of several tests, WG-SS move away from using the term disability, as it is often understood differently across cultures and is sometimes perceived as stigmatizing [7–9]. Upcoming evidence shows that the WG-SS is an efficient tool to identify persons with disabilities at a population level, the results from its implementation allows disaggregation by functional difficulty and severity and enables analysis of inter-regional differences in disability prevalence [10]. The adoption of the WG-SS is growing but still limited. As their implementation grows, so does the understanding of the collected data, its reach, and limitations.

Ensuring health equity is at the core of achieving Sustainable Development Goal 3 of the United Nations and accomplishing Universal Health Coverage. Therefore, getting insights into health-inequities is timely, especially in LMICs, where nearly 80% of persons with disabilities live [1]. One hundred sixty-four countries around the globe are signatories to the Conventions on the Rights of Persons with Disabilities (CRPD) [11]. The CRPD intended to protect the rights and dignity of persons with disabilities. Quality internationally comparable disability disaggregated data are the core of the implementation of the rights of persons with disabilities. CRPD article 31 mandates collecting appropriate information, including statistical and research data. This reinforced the Inclusive Data Charter at the core of the Sustainable Development Goals.

Since there is limited published research in the context of LMICs focussing on the associations between different types of functional difficulties, we aim to fill this research gap. Such research is a first necessary step to find out whether a functional difficulty in one domain (e.g. hearing) may be related to a loss of functioning in another domain (e.g. cognition). In this analysis, we do not intend to infer causality.

## Methods

### Study design and settings

We used nationally representative population census data from six countries: Mauritius (year = 2011; *n* = 988,060), Morocco (year = 2014; *n* = 23,983,300), Senegal (year = 2013; *n* = 7,288,742), Myanmar (year = 2014; *n* = 35,894,738), Vietnam (year = 2009; *n* = 64,267,057), and Uruguay (year = 2011; *n* = 2,478,630). Only countries that have used the internationally comparable questions on disability aligned with the WG-SS as recommended by the United Nations Principles and Recommendations for Population and Housing Censuses were included [12]. Note that a binary screener was used before administration of the functional difficulties questions one dataset - Mauritius. More details on the disability questions for each country are presented in Table S1. Datasets were retrieved from the IPUMS website (https://international.ipums.org/international/).

#### Patient and public involvement

Neither patients nor the public were involved in the design, conduct, reporting, or dissemination plans of our research.

### Variables used

For each country, we considered functional difficulties at the individual level along with demographic characteristics: age, sex (males/females) and location (urban/rural). In this study, we included only adults (aged 18 or more). We did not have any incomplete records in our datasets.

### Statistical analyses

We report the weighted estimates and weighted percentages that are representative of the whole country’s population, by incorporating sample weights in our analyses.

#### Regression

To get an insight into how having a functional difficulty in one domain and controlling for age, sex, and location, could be correlated with having functional difficulties in other domains, we used logistic regression: the response variable could take only binary values (“difficulty”/“no difficulty”).

Correlations among all the predictors - age, location, sex, and all the functional difficulty-related variables (*‘pred_see’, ‘pred_hear’, ‘pred_care’, ‘pred_comm’, ‘pred_mob’, and ‘pred_cogn’*) were computed. There was a strong positive correlation (using 0.6 as the threshold) among all the functional difficulty-related variables (pred_*). This explains the motivation behind considering only one functional difficulty-related variable at a time, in our analysis. There was no or a weak correlation (i.e., below a particular threshold of 0.6; if any), among the control variables - age, sex, and location.

A functional difficulty in a given domain (say seeing) is a binary variable taking the value of 0 for “no difficulty” and 1 for “any level of difficulty” (“unable to do” or “a lot of difficulty” or “some difficulty”). Each functional difficulty variable in a given domain is used in turn as a response variable and a predictor. For instance, when seeing is a response variable, hearing, mobility, cognition, communication, and self-care, are predictors. The term ‘core’ encompasses hearing, seeing, mobility, and cognition. Functional difficulty data corresponding to the four core domains is available for all six countries. However, only Mauritius, Morocco, and Senegal, have data for two more domains - communication and self-care. Other predictors include age, sex, and location. Age is a continuous variable while the other two predictors (sex and location) are binary variables.

We report the weighted odds ratios (ORs) obtained from the logistic regression, as they are widely used to compare two groups (living with a functional difficulty in a domain or not).

Logistic regression models look like

$$res\_* = pred\_* + sex + age + location$$

Country-wise ORs are obtained using the equation given below:

$$ORs = exp(coefficient\, of \,pred\_*)$$

We have specified the 95% CIs and *p*-values corresponding to each ORs obtained. We also report the crude-estimates (obtained using the model: $res\_* = pred\_*$) of each ORs obtained.

R software (version 4.2.1) was used to perform all the data and statistical analyses. Moreover, age-specific (‘18–44’ years and ‘45+’ years) ORs were also computed.

We also computed cross-country correlations by using the country-specific population weights (refer to Table 2A). We used weights based on the population sizes for adults estimated by the UN (https://population.un.org/wpp/).

### Sensitivity analyses

We used different thresholds for the functional difficulty variables to assess if results may vary when the threshold is higher so that it focuses on more severe functional difficulties. We considered two other thresholds as follows.

- First threshold: A functional difficulty in a given domain was assigned 0 if people responded with either “no difficulty” or “some difficulty” and 1 if people responded with either “a lot of difficulty” or “unable to do” to the disability questions asked.
- Second threshold: A functional difficulty in a given domain was assigned 0 if people responded with “no difficulty” or “some difficulty” or “a lot of difficulty” and 1 if people responded with “unable to do” to the disability questions asked.

## Results

### Sample characteristics of the data included

Table 1 lays out gender, location, and the proportion of people reporting experiencing “any level of functional difficulties” in each country. This table only provides a general overview and findings are not statistically tested at this point. More women were present in the sample for each country. Their proportions ranged from 51% in Mauritius, Morocco, and Vietnam to 54% in Myanmar. Proportion of the population reported having “any level of difficulty” in at least one domain in both sexes ranged from 4.8% in Mauritius to 22.1% in Uruguay. Moreover, this proportion was higher among women in all six countries.

**Table 1:** Sample characteristics of the country-wise census data included in this study. We did not perform any statistical test here

**Country**	**Variables**	**All population (%)**	**Population reporting having “any level of difficulty” in at least one domain (weighted)**
Mauritius	***Sex:***		
	Men	484,040 (48.99%)	23,060 (4.76%)
	Women	504,020 (51.01%)	26,280 (5.21%)
	***Location:***		
	Urban	407,380 (41.23%)	20,260 (4.97%)
	Rural	580,680 (58.77%)	29,080 (5.01%)
Morocco	***Sex:***		
	Men	11,811,330 (49.32%)	1,636,460 (13.86%)
	Women	12,135,380 (50.68%)	1,807,290 (14.89%)
	***Location:***		
	Urban	14,909,130 (62.26%)	2,140,210 (14.36%)
	Rural	9,037,580 (37.74%)	1,303,540 (14.42%)
Senegal	***Sex:***		
	Men	3,503,319 (48.06%)	265,616 (7.58%)
	Women	3,785,422 (51.94%)	319,553 (8.44%)
	***Location:***		
	Urban	3,665,779 (50.29%)	278,603 (7.60%)
	Rural	3,622,963 (49.71%)	306,566 (8.46%)
Myanmar	***Sex:***		
	Men	15,195,263 (45.75%)	881,281 (5.80%)
	Women	18,021,015 (54.25%)	1,116,624 (6.20%)
	***Location:***		
	Urban	10,321,327 (31.07%)	468,453 (4.54%)
	Rural	22,894,951 (68.93%)	1,529,453 (6.68%)
Vietnam	***Sex:***		
	Men	31,234,368 (48.6%)	2,604,922 (8.34%)
	Women	33,032,689 (51.4%)	3,272,438 (9.91%)
	***Location:***		
	Urban	19,709,753 (30.67%)	1,425,619 (7.23%)
	Rural	44,557,303 (69.33%)	4,451,741 (9.99%)
Uruguay	***Sex:***		
	Men	1,165,160 (47.01%)	188,770 (16.20%)
	Women	1,313,470 (52.99%)	290,700 (22.13%)

No information about the residents’ location was available for Uruguay. Among three out of five countries (Mauritius, Myanmar, Vietnam) with the information available about location, more rural residents were present in the sample. Their proportions ranged from 38% in Morocco to 69% in Myanmar and Vietnam. The proportion of the population that reported having “any level of difficulty” in at least one domain among rural and urban residents, ranged from 4.5% in Myanmar to 14.4% in Morocco. Moreover, this proportion was higher among rural residents in all five countries.

### Prevalence of ‘any level of difficulty’ in various functional domains

#### Variation across functional domains (country-wise estimates)

**Fig. 1:**
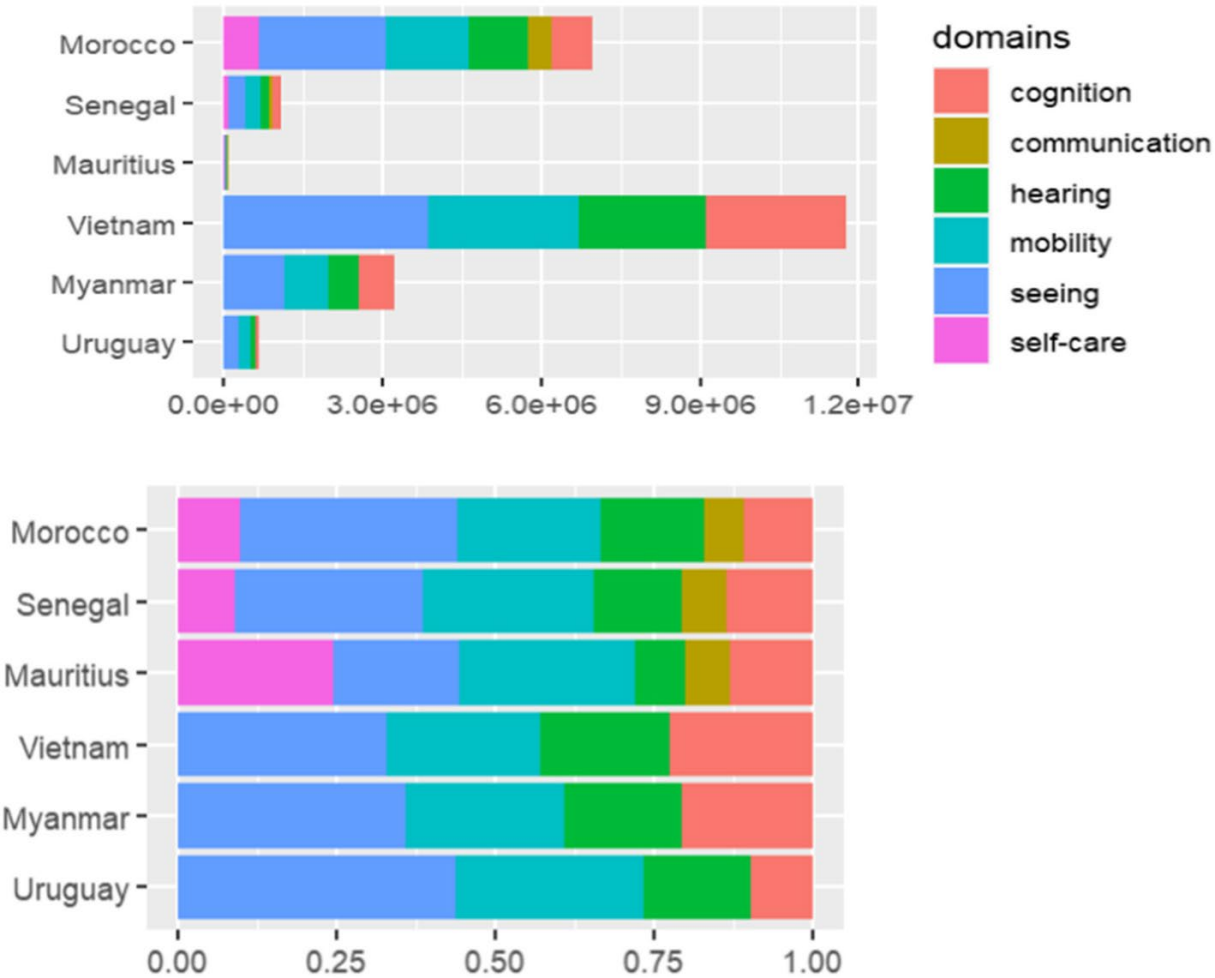
Prevalence of ‘any level of difficulty’ by functional domain (both non-100% stacked and 100% stacked), for each country

Prevalence was the highest for the seeing domain, in five out of six countries included (Uruguay: 11.94%, Morocco: 9.96%, Vietnam: 6.00%, Senegal: 4.35%, Myanmar: 3.48%). In Mauritius, the maximum prevalence was in the mobility domain - 2.46%. Across the functional domains, for three out of six countries (Morocco - 1.81%, Senegal - 1.06%, Mauritius - 0.63%), the lowest prevalence corresponded to the communication domain; for two out of six countries (Vietnam - 3.73% and Myanmar - 1.79%), the lowest prevalence corresponded to the hearing domain (Fig. 1).

#### Variation across countries (functional domain-wise estimates)

Prevalence across the countries ranged from 1.76% in Mauritius to 11.94% in Uruguay in the seeing domain, 0.71% in Mauritius to 4.73% in Morocco in the hearing domain, 2.43% in Myanmar to 8.07% in Uruguay in the mobility domain, 1.14% in Mauritius to 4.14% in Vietnam corresponding to the cognition domain, 0.63% in Mauritius to 1.81% in Morocco in the communication domain, and 1.33% in Senegal to 2.84% in Morocco in the self-care domain (Fig. 1).

### Overall ORs/cross-country estimates

We computed the ORs using the master dataset obtained after combining the data from all six countries. ORs corresponding to each of the four core functional domains were greater than one and all the corresponding *p*-values were < 0.001, suggesting significant positive correlations between the functional difficulties across these domains. The pairwise correlations between domains - i) hearing and cognition, and ii) mobility and cognition, were the highest (Table 2B).

**Table 2:** Overall ORs/cross-country estimates

A. Population weights computed for all six countries.				
**Countries**	**Year**	**Sample sizes of datasets considered (n1)**	**UN Population Estimates (adults) (n2)**	**Population weights** **Computed (= n2/n1)**
Mauritius	2011	988,060	1,008,000	1.020180961
Morocco	2014	23,983,300	24,612,000	1.026214074
Senegal	2013	7,288,742	7,712,000	1.058070103
Myanmar	2014	35,894,738	37,332,000	1.040041022
Vietnam	2009	64,267,057	65,101,000	1.012976213
Uruguay	2011	2,478,630	2,624,000	1.058649335
B. Combined multi-country ORs: Overall ORs averaged out over the six countries along with their 95% CIs, corresponding to each core domain (hearing, seeing, mobility, cognition), using the population weights computed. All the *p*-values were < 0.001 corresponding to each odds ratio implying statistically significant results at the 5% threshold.				
**Predictors →** **Response var ↓**	**Hearing**	**Seeing**	**Mobility**	**Cognition**
Hearing	-	*15.4 (15.2, 15.7)*	13.0 (12.8, 13.2)	22.5 (22.2, 22.9)
Seeing	15.3 (15.0, 15.6)	-	10.2 (10.1, 10.3)	12.3 (12.1, 12.5)
Mobility	12.8 (12.6, 13.0)	10.3 (10.2, 10.5)	-	*29.0 (28.5, 29.4)*
Cognition	*23.6 (23.2, 24.0)*	12.6 (12.4, 12.8)	*29.4 (29.0, 29.9)*	-

### ORs corresponding to the four core functional domains among all countries included

In all six countries, ORs are consistently greater than one, overall (all age-groups combined), and in both age-groups: ‘18–44’ and ‘45+’, and the *p*-values corresponding to each ORs were < 0.001 (Tables 3 and 4 and Table S2). This implies significant positive correlations between the functional difficulties across the four core functional domains. Having a functional difficulty in a given domain (say seeing) is consistently significantly correlated with having a functional difficulty in each of the other domains (hearing, mobility, and cognition). Across countries, Uruguay shows the lowest ORs, across all domains. For instance, ORs corresponding to the hearing-predictor are 6.3 as compared to 16.0 in Morocco, 23.1 in Mauritius, 24.1 in Myanmar, 25.3 in Senegal, and 42.6 in Vietnam, in the cognition domain.

**Table 3:** Main analysis (estimates in the presence of control variables; adjusted ORs estimates): Odds ratios of having a difficulty in a given domain (e.g. hearing) given a difficulty in another domain (e.g. seeing), along with their 95% CIs. All the *p*-values were < 0.001 corresponding to each odds ratio implying statistically significant results at the 5% threshold. 988,060, 23,983,300, 7,288,742, 35,894,738, 64,267,057, 2,478,630, were the cell counts used in the computation of ORs for Mauritius, Morocco, Senegal, Myanmar, Vietnam, and Uruguay respectively

**Predictors →** **Response var ↓**	**Hearing**	**Seeing**	**Mobility**	**Cognition**
***Mauritius***				
Hearing	-	22.6 (21.4, 23.9)	12.8 (12.1, 13.5)	20.0 (18.8, 21.3)
Seeing	21.9 (20.7, 23.2)	-	12.7 (12.2. 13.2)	14.0 (13.3, 14.7)
Mobility	12.2 (11.5, 12.9)	12.8 (12.3, 13.3)	-	28.3 (26.9, 29.7)
Cognition	23.1 (21.7, 24.5)	15.5 (14.8, 16.3)	29.5 (28.1, 30.9)	-
***Morocco***				
Hearing	-	14.4 (14.4, 14.5)	10.8 (10.7, 10.9)	15.1 (15.0, 15.2)
Seeing	14.8 (14.8, 14.9)	-	8.7 (8.7, 8.8)	8.8 (8.7, 8.8)
Mobility	10.7 (10.7, 10.8)	8.7 (8.6, 8.7)	-	27.1 (26.9, 27.3)
Cognition	16.0 (15.9, 16.1)	8.7 (8.7, 8.8)	27.0 (26.8, 27.2)	-
***Senegal***				
Hearing	-	17.4 (17.2, 17.6)	19.0 (18.7, 19.2)	25.4 (25.0, 25.7)
Seeing	17.5 (17.2, 17.7)	-	11.2 (11.1, 11.3)	10.1 (10.0, 10.2)
Mobility	18.9 (18.7, 19.2)	11.5 (11.3, 11.6)	-	28.4 (28.0, 28.8)
Cognition	25.3 (24.9, 25.6)	10.3 (10.2, 10.4)	27.9 (27.5, 28.3)	-
***Myanmar***				
Hearing	-	14.4 (14.3, 14.5)	12.1 (12.0, 12.1)	23.5 (23.4, 23.7)
Seeing	14.0 (14.0, 14.1)	-	11.5 (11.4, 11.5)	17.1 (17.0, 17.3)
Mobility	11.9 (11.9, 12.0)	11.8 (11.7, 11.9)	-	46.5 (46.2, 46.8)
Cognition	24.1 (23.9, 24.2)	17.2 (17.1, 17.3)	47.3 (47.0, 47.6)	-
***Vietnam***				
Hearing	-	17.1 (17.1, 17.2)	17.6 (17.5, 17.6)	41.8 (41.7, 42.0)
Seeing	17.2 (17.1, 17.2)	-	11.8 (11.8, 11.9)	18.1 (18.0, 18.1)
Mobility	17.4 (17.3, 17.4)	11.9 (11.9, 12.0)	-	29.0 (28.9, 29.1)
Cognition	42.6 (42.4, 42.8)	17.8 (17.8, 17.9)	29.6 (29.5, 29.7)	-
***Uruguay***				
Hearing	-	3.8 (3.7, 3.8)	3.1 (3.1, 3.2)	5.4 (5.2, 5.5)
Seeing	3.6 (3.6, 3.7)	-	3.3 (3.2, 3.3)	3.6 (3.6, 3.7)
Mobility	3.0 (3.0, 3.1)	3.4 (3.4, 3.5)	-	8.9 (8.7, 9.1)
Cognition	6.3 (6.1, 6.4)	3.8 (3.8, 3.9)	9.6 (9.4, 9.8)	-

Each logistic regression adjusts for sex, age and residence (rural/urban)

**Table 4:** Main analysis (estimates in the presence of control variables; adjusted ORs estimates): Odds ratios of having a difficulty in a given domain (e.g. hearing) given a difficulty in another domain (communication or self-care), along with their 95% CIs. Only Mauritius, Morocco, and Senegal, have data available for communication and self-care domains. All the *p*-values were < 0.001 corresponding to each odds ratio implying statistically significant results at the 5% threshold. 988,060, 23,983,300, 7,288,742, 35,894,738, 64,267,057, 2,478,630, were the cell counts used in the computation of ORs for Mauritius, Morocco, Senegal, Myanmar, Vietnam, and Uruguay respectively

**Predictors →** **Response var ↓**	**Communication**	**Self-care**
***Mauritius***		
Hearing	45.3 (42.2, 48.7)	21.4 (20.2, 22.6)
Seeing	12.3 (11.5, 13.1)	20.3 (19.4, 21.1)
Mobility	62.1 (58.1, 66.4)	59.6 (57.3, 62.1)
Cognition	107.2 (101.1, 113.6)	63.5 (60.9, 66.2)
Self-care	85.3 (80.6, 90.3)	-
Communication	-	84.3 (79.7, 89.1)
***Morocco***		
Hearing	33.1 (32.8, 33.4)	11.5 (11.5, 11.6)
Seeing	8.6 (8.5, 8.6)	7.8 (7.7, 7.8)
Mobility	39.4 (39.1, 39.8)	81.0 (80.3, 81.6)
Cognition	222.8 (220.7, 224.8)	77.6 (77.1, 78.1)
Self-care	323.0 (319.7, 326.3)	-
Communication	-	273.3 (270.8, 275.9)
***Senegal***		
Hearing	27.4 (26.9, 28.0)	18.8 (18.5, 19.1)
Seeing	5.9 (5.8, 6.0)	7.9 (7.8, 8.1)
Mobility	17.0 (16.7, 17.3)	55.9 (54.9, 56.9)
Cognition	66.5 (65.3, 67.8)	65.4 (64.4, 66.5)
Self-care	78.2 (76.7, 79.7)	-
Communication	-	82.1 (80.6, 83.7)

Each logistic regression adjusts for sex, age and residence (rural/urban)

#### ORs for the hearing-predictor

In all six countries, the highest correlation is found between having a functional difficulty in the hearing domain and having a difficulty in the cognition domain (Table 3).

##### Age-specific ORs

A similar pattern is observed within age groups, in the older population - ‘45+’ age-group and in the ‘18–44’ age-group, with the highest correlation between hearing and cognition domains found in most countries (Table S2).

#### ORs for the seeing-predictor

In three among six countries, having a functional difficulty in the seeing domain is most highly correlated with having a difficulty in the hearing domain (Table 3).

##### Age-specific ORs

Similar patterns are seen in the older population - ‘45+’ age-group (highest ORs in the hearing domain in four countries). In the ‘18–44’ age-group also, highest ORs are observed in the hearing domain in all countries (Table S2).

#### ORs for the mobility-predictor

In all six countries, having a functional difficulty in the mobility domain is the most highly correlated with having a difficulty in the cognition domain (Table 3).

##### Age-specific ORs

Similar patterns are observed in the older population - ‘45+’ age-group (highest ORs in the cognition domain in all countries). Also, in the ‘18–44’ age-group, similar patterns are seen in the majority of the countries (highest ORs in the cognition domain in five countries; Table S2).

#### ORs for the cognition-predictor

In five among six countries, having a functional difficulty in the cognition domain is the most highly correlated with having a difficulty in the mobility domain (Table 3).

##### Age-specific ORs

Similar patterns are seen in the older population - ‘45+’ age-group (highest ORs in the mobility domain in five countries). In the ‘18–44’ age-group also, the highest ORs are observed in the mobility domain in four countries. Please refer to Table S2 for more information.

### ORs corresponding to the other two functional domains (communication and self-care) in Mauritius, Morocco, and Senegal

Only three out of the six countries - Mauritius, Morocco, and Senegal, have the data corresponding to the communication and self-care domains.

#### ORs for the communication-predictor

In Senegal and Morocco, having a functional difficulty in the communication domain is the most highly correlated with having a difficulty in the self-care domain (Table 4).

##### Age-specific ORs

Similar patterns are observed in the older population - ‘45+’ age-group (highest ORs in the self-care domain in three countries). In the ‘18–44’ age-group also, a similar pattern is seen (highest ORs in the self-care domain in two countries; Table S2).

#### ORs for the self-care-predictor

In all three countries, having a functional difficulty in the self-care domain is the most highly correlated with having a difficulty in the communication domain (Table 4).

##### Age-specific ORs

Similar patterns are seen in the older population - ‘45+’ age-group (highest ORs in the communication domain in all three countries; Table S2).

### Sensitivity analyses

For both the other thresholds used to define the functional difficulty variables, ORs were greater than one, overall (all age-groups combined), and in both age-groups: ‘18–44’ and ‘45+’, for each country, and the *p*-values corresponding to each ORs were < 0.001. This implies the significant positive correlations between the functional difficulties across all six domains for all the countries.

Similar to the main analyses, having a functional difficulty in the hearing-, seeing-, mobility, cognition-domains are the most highly correlated with having a difficulty in the cognition-, hearing-, cognition-, and mobility-domains, respectively, for the other two thresholds (Tables S3 and S4).

Crude estimates (in the absence of control variables - age, sex, and location) corresponding to each ORs are provided in the Supplementary information (Tables S5-S9).

## Discussion

### Importance of our study

In this study, we compute the correlations between functional difficulties across various domains adjusting for age, sex, and location (urban/rural), using population census data for six countries. We find that the functional difficulties in one domain are significantly positively correlated with those in the other five domains, overall (for all the age-groups combined), for each country. Moreover, age-specific ORs for the two age-groups - ‘18–44’ and ‘45+’ are more than one, suggesting significant positive correlations in both young and older populations, in all countries.

All the correlations found are consistently larger than 1 and statistically significant across all six functional domains, for each country. There exists evidence of disproportionate risks for multimorbidity for persons with disabilities [1], but little information on how functional difficulties may co-occur among persons with disabilities. Our study helps shed light on this by showing that people with functional limitations in one domain (e.g. seeing) are significantly likely to have functional limitations in other domains as well (hearing, mobility, cognition, communication, and self-care), using data with internationally comparable functional difficulty questions from five LMICs - Mauritius, Morocco, Senegal, Myanmar, Vietnam and one high-income country - Uruguay.

Among people having functional limitations in hearing- and mobility-domains, the highest positive correlations were observed with the cognition-domain. These patterns emerge in both main and the analysis with different levels of severity - sensitivity analyses. Moreover, as the level of severity increases (first threshold to second threshold), the odds of someone with limitations in X (say hearing) having limitations in Y (cognition) also increases. In this work, we can only infer associations and not establish causality.

#### Hearing- and cognition-domains

Our study reports that among people having functional limitations in hearing, the highest positive correlation was with cognition, in all countries. Dalal et al. also observed a similar significant positive association in the Colombian context [5]. Moreover, our findings are consistent with the several studies reporting an association between hearing loss/impairment with higher risks of cognitive decline, especially in older adults [13–16]. In another study conducted in China, hearing impairments were associated with higher risks of cognitive decline among middle-aged and older adults [17]. We observe this strong significant positive correlation/association not only in older (‘45+’) but also in the younger (‘18–44’) population as also observed by Dalal et al. in all three age-groups (‘0–14’, ‘15–64’, ‘65+’) considered [5]. Additionally, a study conducted by Croll et al. reported the association between the hearing loss/impairment with higher risks of cognitive decline in the general population [18]. The link between hearing and cognition functional difficulties could be attributed to the fact that sound processing and cognitive processing occur in the same areas of the brain. In fact, the existing studies seem to give a hint on the causal link between the hearing loss and cognitive impairment [19]. This is an active area, and more research is needed to consider hearing loss as a risk factor for cognitive decline. This is out of scope of this work.

#### Mobility- and cognition-domains

Our analyses suggest that among people with functional limitations in mobility, the highest positive correlation was with cognition, overall, and in both younger (‘18–44’) and older (‘45+’) population groups. Tolea et al. assessed the relationship between mobility and cognitive performance, concluding that the likelihood of poor global cognition increases with progression of mobility dysfunction [20]. In a Singapore Longitudinal Ageing Study, Ng et al. investigated the Timed-Up-and-Go (TUG) measure of functional mobility in predicting cognitive decline. The TUG seemed to be accurate in predicting the future risks of serious cognitive outcomes [21]. Moreover, Demnitz et al. studied the relationship between cognitive processes and various aspects of mobility, in the Canadian population aged 45 or more. They demonstrated that all cognitive measures were related to mobility, suggesting a global association. These associations were also present after accounting for multiple confounders related to age, and they tend to increase with age [22].

We also observe that among people with functional limitations in cognition, the highest positive correlation was with mobility. Buchman et al. demonstrated the associations between cognitive function and the risk of incident mobility impairments in older adults, in a prospective, observational cohort study conducted in the USA [23]. In another interesting study, Handling et al. investigated cognitive function as a risk factor for major mobility disability (MMD), showed that in the adjusted model, a positive change in processing speed (a sub-domain of cognitive function) was significantly associated with reduced risk of MMD [24].

Correlations found between the functional difficulties across domains could be attributed to the healthcare-inequities present, environmental factors, cultural factors, and socioeconomic factors. Limited or delayed access to health services is a major factor contributing to higher morbidity among persons with disabilities as compared to their counterparts [1]. In addition, not having access to health facilities affects the person with disabilities more as compared to those without disabilities. Limited ability of the caregivers or health professionals to provide a proper diagnosis is another important factor responsible for an increased risk of poor oral health among people with disabilities alongside their poorer socioeconomic status [25]. This risk becomes higher among those with multiple impairments [26]. Various environmental factors seem to be hindering and limiting the functioning among people with disabilities, more than their counterparts, especially in terms of their daily activities and participation in society. Inaccessible or unaffordable transportation restricts persons with disabilities, and having a limited number of places to socialize makes it hard for persons with disabilities to engage in any community activities [1].

Uruguay, the only high-income country, reports the lowest correlations (as depicted by the lowest ORs), corresponding to the predictors for four functional core domains, among all the countries included in this study. Therefore, there exists the need to consider people with multiple functional difficulties, especially in studies on socioeconomic outcomes (e.g. poverty, employment) given how frequent having multiple functional difficulties is [1, 3]. This further emphasizes the importance of capturing the right estimates of people having multiple functional difficulties so as to come up with suitable interventions for them.

Further research investigating the high pairwise correlations present between functional difficulties across the domains - hearing & cognition, mobility & cognition, is needed, to get better insights into these associations.

### Strengths and limitations

To our knowledge, this is the first study to explicitly study the correlations between functional difficulties across various functional domains, using the national census data from six countries. Dalal et al. only studied the odds of someone with hearing limitations also having limitations in- seeing, mobility, cognition, self-care, and communication [5]. Our results are very robust irrespective of the different age-groups and thresholds for predictors and response variables considered. However, we included only the adult population in this study. Very little disability-disaggregated data is available worldwide. We believe that our study contributes to providing more visibility to the population at risk - people with functional difficulties, by gaining insights into the extent of health-inequities faced by them as compared to the rest of the population. This would help in coming up with the suitable recommendations/policies to help them.

Our study has limitations. In our analysis, we consider only the two categories/groups for both predictors and response variables (“difficulty” or “no difficulty”). However, we have considered two other thresholds in the sensitivity analyses. This could be extended by considering four categories for both predictors and response variables (“no difficulty”, “some difficulty”, “a lot of difficulty”, “unable to do”). Moreover, we could include only six countries in the analysis limited by the availability of countries that have used the internationally comparable questions on disability as recommended by the United Nations Principles and Recommendations for Population and Housing Censuses ([10], p. 207). While such census and national survey data allow for detailed epidemiological analyses, they do not adequately capture persons with psychosocial disabilities. Improving data quality, quantity and availability shall enable the rolling out of the CRPD [11] as it would help us gain insights into the right disability-prevalence estimates.

### Conclusions

This study reports the consistent positive associations in the experience of functional difficulties in various domains in the six countries under study. This is the first analysis focussing on the correlations between different types of functional difficulties in the context of LMICs, using national census data. This study highlights the importance of the availability of disability data across countries. The large correlations found in functional difficulties across domains may reflect barriers to health care services and resources (e.g. assistive devices) that may lead to an avoidable deterioration of functioning across domains. Further research is needed on the trajectories of functional difficulties over time and on structural barriers that people with functional difficulties may experience in their communities and in healthcare settings in particular. This is important as some functional difficulties may be preventable and persons with disabilities have the right to the highest attainable standard of health.

### Supplementary Information

**Additional file 1: Table S1.** An overview of the datasets used in the analysis. **Table S2.** Main analysis (adjusted estimates): Age-specific odds ratios in the presence of all control variables. **Table S3.** Sensitivity analysis (adjusted estimates) - first threshold: Overall odds ratios in the presence of all control variables. **Table S4.** Sensitivity analysis (adjusted estimates) - second threshold: Overall odds ratios in the presence of all control variables. **Table S5.** Main analysis (crude estimates): Odds ratios of having a difficulty in a given domain (e.g. hearing) given a difficulty in another domain (e.g. seeing). **Table S6.** Main analysis (crude estimates): Odds ratios of having a difficulty in a given domain (e.g. hearing) given a difficulty in another domain (communication or self-care). **Table S7.** Main analysis (crude estimates): Age-specific odds ratios in the presence of all control variables. **Table S8.** Sensitivity analysis (crude estimates) - first threshold: Overall odds ratios in the presence of all control variables. **Table S9.** Sensitivity analysis (crude estimates) - second threshold: Overall odds ratios in the presence of all control variables.

## Data Availability

We used the national census data that is publicly available. Datasets were retrieved from the IPUMS website (https://international.ipums.org/international/).
