# Supplementary material for: Links across disabilities: unveiling associations between functional domains
Source: BMC Public Health. 2024 Jan 2;24:41. doi: 10.1186/s12889-023-17523-5 (PMC10763216; doi:10.1186/s12889-023-17523-5)
Supplement: Supplementary file 1 — Additional file 1: Table S1. An overview of the datasets used in the analysis. Table S2. Main analysis (adjusted estimates): Age-specific odds ratios in the presence of all control variables. Table S3. Sensitivity analysis (adjusted estimates) - first threshold: Overall odds ratios in the presence of all control variables. Table S4. Sensitivity analysis (adjusted estimates) - second threshold: Overall odds ratios in the presence of all control variables. Table S5. Main analysis (crude estimates): Odds ratios of having a difficulty in a given domain (e.g. hearing) given a difficulty in another domain (e.g. seeing). Table S6. Main analysis (crude estimates): Odds ratios of having a difficulty in a given domain (e.g. hearing) given a difficulty in another domain (communication or self-care). Table S7. Main analysis (crude estimates): Age-specific odds ratios in the presence of all control variables. Table S8. Sensitivity analysis (crude estimates) - first threshold: Overall odds ratios in the presence of all control variables. Table S9. Sensitivity analysis (crude estimates) - second threshold: Overall odds ratios in the presence of all control variables. [file 12889_2023_17523_MOESM1_ESM.pdf]

Supplementary Information (SI)

Links across disabilities: Unveiling associations between functional domains

Jyoti Dalal, Sophie Mitra, Ananthu James, Minerva Rivas Velarde

Table of Contents

Table S1: *An overview of the datasets used in the analysis.*
Table S2: *Main analysis (adjusted estimates): Age-specific odds ratios in the presence of all control variables*
Table S3: *Sensitivity analysis (adjusted estimates) - first threshold: Overall odds ratios in the presence of all control variables*
Table S4: *Sensitivity analysis (adjusted estimates) - second threshold: Overall odds ratios in the presence of all control variables*
Table S5: *Main analysis (crude estimates): Odds ratios of having a difficulty in a given domain (e.g. hearing) given a difficulty in another domain (e.g. seeing)*
Table S6: *Main analysis (crude estimates): Odds ratios of having a difficulty in a given domain (e.g. hearing) given a difficulty in another domain (communication or self-care)*
Table S7: *Main analysis (crude estimates): Age-specific odds ratios in the presence of all control variables*
Table S8: *Sensitivity analysis (crude estimates) - first threshold: Overall odds ratios in the presence of all control variables*
Table S9: *Sensitivity analysis (crude estimates) - second threshold: Overall odds ratios in the presence of all control variables*

$$Functional\ domain\ y = Functional\ domain\ x + sex + location + age,$$

where x = one among the hearing, seeing, mobility, cognition, communication, and self-care domains.

For instance, if the aim is to understand how having functional difficulties ('difficulty') in the hearing-domain could be correlated with having functional difficulties in the seeing- and mobility-domains, then the resulting regression equations look like:

$$res\_see = pred\_hear + sex + age + location,$$
$$res\_mob = pred\_hear + sex + age + location,\ respectively.$$

Table S1: An overview of the datasets used in the analysis.

**Countries included**	**Year**	**Sample sizes**	**Disability questions**
Mauritius	2011	988,060	State if the person has any difficulty to perform a daily-life activity considered normal for his/her age.<br><br>If **YES**, state the severity of all applicable difficulties as follows: 1. Some difficulty 2. A lot of difficulty 3. Cannot do at all<br><br>- Seeing even if wearing glasses<br>- Hearing even if using a hearing aid<br>- Walking or climbing stairs<br>- Remembering, concentrating or acquiring education and learning<br>- Looking after oneself with regard to feeding, personal care and hygiene<br>- Speaking and talking
Morocco	2014	23,983,300	Préciser le degré de difficulté qu'a la personne à<br>- voir<br>- entendre<br>- marcher ou monter l'escalier<br>- se rappeler ou se concentrer<br>- prendre soin de soi comme prendre un bain ou s'habiller<br>- communiquer dans sa langue habituelle
Senegal	2013	7,288,742	- [NOM] a-t-il des difficultés à voir, même s'il porte des

			lunettes?<br>- [NOM] a-t-il des difficulté à entendre, même à l'aide d'une prothèse auditive?<br>- [NOM] a-t-il des difficultés à marcher ou à monter des marches?<br>- [NOM] a-t-il du mal à se souvenir ou à se concentrer?<br>- [NOM] a-t-il des difficultés (en prenant soin de luimême) à effectuer des activités comme se laver?<br>- En utilisant sa langue habituelle (usuelle), [NOM] a-t-il des difficultés à communiquer, (par exemple à comprendre ou se faire comprendre par les autres)?
Myanmar	2014	35,894,738	Does (Name) have any difficulty...?<br><br>- Seeing, even if wearing glasses<br>- Hearing, even if using hearing aid<br>- Walking, climbing steps, carrying items<br>- Remembering or concentrating
Vietnam	2009	64,267,057	Has [NAME] has any difficulty as:<br><br>- Seeing (even if wearing glasses)?<br>- Hearing?<br>- Walking?<br>- Remembering or paying attention to?
Uruguay	2011	2,478,630	Tiene… (Nombre) dificultad permanente para…<br><br>- Ver, aun si usa anteojos o lentes?<br>- Oír, aun si usa audífono?<br>- Caminar o subir escalones?<br>- Entender y/o aprender?

Part 1: Adjusted ORs estimates

Age-specific estimates in the presence of control variables:

Table S2: *Main analysis (adjusted estimates):* Age-specific odds ratios in the presence of all control variables, along with their 95% CIs. All the p-values were <0.001 corresponding to each

odd ratio implying statistically significant results at the 5% threshold.

1. 45+ age-group:

ORs corresponding to the four core functional domains among all countries included (n = 6):

**Predictors →**<br><br>**Response var ↓**	**Hearing**	**Seeing**	**Mobility**	**Cognition**
Mauritius				
Hearing	-	19.1 (18.0, 20.2)	11.1 (10.5, 11.8)	16.4 (15.3, 17.5)
Seeing	18.9 (17.8, 20.0)	-	11.9 (11.4, 12.3)	12.2 (11.5, 12.8)
Mobility	11.0 (10.3, 11.6)	11.9 (11.4, 12.4)	-	21.3 (20.2, 22.5)
Cognition	17.0 (15.9, 18.1)	12.4 (11.7, 13.0)	21.3 (20.2, 22.4)	-
Morocco				
Hearing	-	13.1 (13.0, 13.1)	8.7 (8.7, 8.8)	11.2 (11.1, 11.3)
Seeing	13.2 (13.1, 13.2)	-	8.0 (8.0, 8.0)	7.6 (7.5, 7.6)
Mobility	8.7 (8.7, 8.8)	8.0 (7.9, 8.0)	-	18.7 (18.6, 18.9)
Cognition	11.2 (11.1, 11.3)	7.5 (7.4, 7.5)	18.4 (18.3, 18.5)	-
Senegal				
Hearing	-	14.6 (14.4, 14.9)	14.6 (14.4, 14.8)	17.8 (17.5, 18.1)
Seeing	14.5 (14.3, 14.7)	-	9.9 (9.8, 10.1)	8.9 (8.7, 9.0)
Mobility	14.6 (14.3,	10.0 (9.9, 10.1)	-	21.3 (21.0, 21.6)

	14.8)			
Cognition	17.8 (17.5, 18.0)	9.0 (8.9, 9.2)	21.3 (20.9, 21.6)	-

Myanmar				
Hearing	-	13.7 (13.7, 13.8)	10.7 (10.6, 10.8)	19.4 (19.2, 19.5)
Seeing	13.6 (13.5, 13.7)	-	11.0 (10.9, 11.0)	16.1 (16.0, 16.2)
Mobility	10.7 (10.6, 10.7)	11.0 (11.0, 11.1)	-	40.1 (39.8, 40.4)
Cognition	19.3 (19.2, 19.4)	16.1 (16.0, 16.2)	40.1 (39.8, 40.4)	-
Vietnam				
Hearing	-	14.4 (14.4, 14.5)	13.3 (13.3, 13.4)	28.5 (28.4, 28.6)
Seeing	14.2 (14.2, 14.3)	-	10.2 (10.1, 10.2)	15.4 (15.4, 15.5)
Mobility	13.3 (13.2, 13.3)	10.3 (10.2, 10.3)	-	21.9 (21.8, 22.0)
Cognition	28.3 (28.2, 28.5)	15.5 (15.4, 15.5)	21.9 (21.8, 22.0)	-
Uruguay				
Hearing	-	3.7 (3.6, 3.7)	2.9 (2.8, 2.9)	4.3 (4.2, 4.4)
Seeing	3.7 (3.6, 3.7)	-	3.3 (3.3, 3.4)	3.5 (3.4, 3.5)
Mobility	2.9 (2.8, 2.9)	3.3 (3.3, 3.4)	-	6.7 (6.6, 6.9)
Cognition	4.4 (4.3, 4.5)	3.5 (3.4, 3.5)	6.7 (6.5, 6.8)	-

ORs corresponding to the two functional domains (communication and self-care) in Mauritius, Morocco, and Senegal.

**Predictors →**	**Communication**	**Self-care**

Response var ↓		
Mauritius		
Hearing	27.2 (25.2, 29.5)	17.3 (16.3, 18.4)
Seeing	9.5 (8.8, 10.2)	14.5 (13.8, 15.2)
Mobility	38.3 (35.5, 41.3)	40.5 (38.6, 42.4)
Cognition	50.0 (46.6, 53.6)	38.4 (36.4, 40.4)
Self-care	61.4 (57.4, 65.7)	-
Communication	-	58.2 (54.3, 62.2)
Morocco		
Hearing	16.2 (16.0, 16.4)	8.1 (8.0, 8.1)
Seeing	6.5 (6.5, 6.6)	6.4 (6.3, 6.4)
Mobility	19.9 (19.7, 20.1)	41.8 (41.4, 42.2)
Cognition	94.9 (93.8, 96.0)	36.8 (36.5, 37.1)
Self-care	144.6 (142.7, 146.4)	-
Communication	-	136.2 (134.6, 137.9)
Senegal		
Hearing	16.7 (16.4, 17.1)	13.3 (13.1, 13.6)
Seeing	5.1 (5.0, 5.2)	6.9 (6.8, 7.0)
Mobility	13.0 (12.7, 13.3)	33.9 (33.1, 34.6)
Cognition	37.3 (36.4, 38.2)	35.8 (35.1, 36.5)
Self-care	48.3 (47.1, 49.4)	-
Communication	-	48.2 (47.1, 49.4)

2. '18-44' age-group

ORs corresponding to the four core functional domains among all countries included (n = 6):

**Predictors →**<br>**Response var ↓**	**Hearing**	**Seeing**	**Mobility**	**Cognition**
Mauritius				
Hearing	-	117.5 (98.7, 140.0)	39.6 (32.4, 48.6)	60.7 (51.0, 72.3)
Seeing	117.5 (98.7, 140.0)	-	34.6 (30.3, 39.6)	34.3 (30.1, 39.1)
Mobility	39.5 (32.2, 48.4)	34.5 (30.1, 39.4)	-	65.4 (59.3, 72.1)
Cognition	60.6 (50.9, 72.2)	34.3 (30.1, 39.1)	64.7 (58.7, 71.3)	-
Morocco				
Hearing	-	25.0 (24.7, 25.3)	30.7 (30.3, 31.1)	39.9 (39.4, 40.4)
Seeing	25.0 (24.7, 25.3)	-	13.8 (13.7, 14.0)	12.6 (12.5, 12.8)
Mobility	30.6 (30.3, 31.0)	13.8 (13.7, 13.9)	-	47.7 (47.2, 48.2)
Cognition	40.0 (39.5, 40.5)	12.6 (12.5, 12.7)	47.7 (47.2, 48.2)	-
Senegal				
Hearing	-	31.1 (30.4, 31.9)	37.6 (36.7, 38.5)	66.6 (64.9, 68.4)
Seeing	31.1 (30.4, 31.9)	-	18.2 (17.8, 18.6)	18.5 (18.1, 19.0)
Mobility	37.4 (36.5, 38.4)	18.1 (17.8, 18.5)	-	48.4 (47.3, 49.6)
Cognition	66.7 (64.9, 68.5)	18.5 (18.0, 19.0)	48.5 (47.3, 49.6)	-
Myanmar				
Hearing	-	28.9 (28.4, 29.4)	29.8 (29.2, 30.3)	64.4 (63.4, 65.5)
Seeing	29.0 (28.5, 29.5)	-	24.6 (24.2, 25.0)	27.4 (26.9, 27.8)
Mobility	29.8 (29.3, 30.4)	24.6 (24.2, 25.0)	-	72.9 (71.9, 73.9)

Cognition	64.5 (63.5, 65.6)	27.6 (27.2, 28.0)	72.9 (71.9, 73.9)	-
Vietnam				
Hearing	-	70.8 (70.2, 71.4)	89.3 (88.5, 90.1)	200.8 (199.0, 202.6)
Seeing	70.2 (69.6, 70.8)	-	43.0 (42.6, 43.4)	44.2 (43.9, 44.6)
Mobility	89.3 (88.4, 90.1)	43.1 (42.8, 43.5)	-	86.6 (85.9, 87.2)
Cognition	200.8 (199.1, 202.6)	44.5 (44.1, 44.8)	86.5 (85.9, 87.2)	-
Uruguay				
Hearing	-	6.0 (5.7, 6.2)	8.6 (8.2, 9.1)	13.5 (12.9, 14.2)
Seeing	6.0 (5.7, 6.2)	-	5.1 (4.9, 5.3)	4.9 (4.7, 5.0)
Mobility	8.6 (8.1, 9.1)	5.1 (4.9, 5.3)	-	18.6 (17.9, 19.4)
Cognition	13.6 (12.9, 14.2)	4.9 (4.7, 5.1)	18.6 (17.9, 19.4)	-

ORs corresponding to the two functional domains (communication and self-care) in Mauritius, Morocco, and Senegal:

**Predictors →**<br>**Response var ↓**	**Communication**	**Self-care**
Mauritius		
Hearing	392.1 (334.3, 459.9)	131.0 (110.2, 155.7)
Seeing	50.8 (43.8, 58.9)	206.5 (184.2, 231.6)
Mobility	131.9 (117.9, 147.6)	521.8 (465.9, 584.5)
Cognition	405.6 (363.9, 452.1)	420.1 (378.5, 466.2)
Self-care	376.4 (332.8, 425.7)	-
Communication	-	459.9 (402.4, 525.7)
Morocco		

Hearing	83.3 (82.2, 84.3)	43.8 (43.2, 44.4)
Seeing	13.2 (13.1, 13.4)	14.8 (14.6, 15.0)
Mobility	60.1 (59.4, 60.8)	143.6 (141.9, 145.4)
Cognition	392.2 (387.0, 397.5)	309.5 (305.6, 313.5)
Self-care	480.8 (474.2, 487.5)	-
Communication	-	480.7 (474.1, 487.4)
Senegal		
Hearing	62.5 (60.6, 64.3)	59.1 (57.3, 61.1)
Seeing	10.7 (10.3, 11.0)	19.2 (18.6, 19.8)
Mobility	24.4 (23.7, 25.2)	108.7 (105.6, 111.9)
Cognition	117.4 (114.2, 120.6)	208.3 (202.3, 214.6)
Self-care	148.5 (144.0, 153.0)	-
Communication	-	148.6 (144.1, 153.1)

Sensitivity analyses:

Country-specific estimates:

- First threshold: A functional difficulty in a given domain was assigned 0 if people responded with either "no difficulty" or "some difficulty" and 1 if people responded with either "a lot of difficulty" or "unable to do" to the disability questions asked.

Table S3: *Sensitivity analysis (adjusted estimates):* Overall odds ratios in the presence of all control variables, along with their 95% CIs. All the p-values were <0.001 corresponding to each odd ratio implying statistically significant results at the 5% threshold. 988060, 23983300, 7288742, 35894738, 64267057, 2478630, were the cell counts used in the computation of ORs for Mauritius, Morocco, Senegal, Myanmar, Vietnam, and Uruguay respectively.

ORs corresponding to the four core functional domains among all countries included (n = 6):

**Predictors →<br><br>Response var ↓**	**Hearing**	**Seeing**	**Mobility**	**Cognition**
Mauritius				
Hearing	-	23.1 (21.1, 25.3)	15.7 (14.4, 17.1)	29.6 (27.1, 32.4)
Seeing	21.0 (19.2, 23.1)	-	15.9 (14.9, 17.0)	15.5 (14.3, 16.8)
Mobility	14.3 (13.2, 15.6)	16.1 (15.2, 17.2)	-	33.6 (31.4, 35.8)
Cognition	35.2 (32.2, 38.3)	19.2 (17.8, 20.7)	37.3 (35.0, 39.7)	-
Morocco				
Hearing	-	19.0 (18.8, 19.1)	11.9 (11.8, 12.0)	24.2 (24.0, 24.4)
Seeing	18.5 (18.4, 18.7)	-	9.4 (9.3, 9.5)	12.6 (12.5, 12.7)
Mobility	11.5 (11.4, 11.5)	9.3 (9.3, 9.4)	-	29.1 (28.8, 29.3)
Cognition	26.7 (26.4, 26.9)	13.9 (13.8, 14.0)	29.8 (29.5, 30.0)	-
Senegal				
Hearing	-	44.8 (43.6, 46.1)	43.7 (42.4, 45.0)	98.1 (95.1, 101.3)
Seeing	44.3 (43.1, 45.6)	-	20.7 (20.2, 21.1)	23.5 (22.8, 24.2)
Mobility	42.3 (41.1, 43.6)	20.5 (20.0, 20.9)	-	70.3 (68.3, 72.3)
Cognition	98.6 (95.6, 101.7)	24.4 (23.7, 25.1)	71.4 (69.5, 73.5)	-
Myanmar				
Hearing	-	41.1 (40.5, 41.8)	27.5 (27.1, 28.0)	76.3 (75.2, 77.5)
Seeing	38.0 (37.4, 38.7)	-	24.2 (23.8, 24.5)	30.9 (30.4, 31.4)
Mobility	26.2 (25.8, 26.6)	25.4 (25.1, 25.8)	-	123.3 (121.7, 124.9)

Cognition	81.1 (79.9, 82.3)	36.9 (36.4, 37.5)	132.7 (131.0, 134.4)	-
Vietnam				
Hearing	-	72.9 (72.3, 73.5)	54.2 (53.8, 54.6)	157.0 (155.8, 158.2)
Seeing	71.1 (70.5, 71.7)	-	52.8 (52.4, 53.2)	64.3 (63.9, 64.8)
Mobility	52.6 (52.2, 53.0)	52.5 (52.2, 52.9)	-	106.5 (105.7, 107.2)
Cognition	167.0 (165.8, 168.3)	70.6 (70.0, 71.1)	112.8 (112.0, 113.6)	-
Uruguay				
Hearing	-	6.0 (5.8, 6.2)	4.8 (4.7, 5.0)	11.3 (10.8,11.8)
Seeing	5.7 (5.6, 5.9)	-	5.5 (5.4, 5.7)	6.6 (6.3, 6.9)
Mobility	4.5 (4.4, 4.7)	5.7 (5.6, 5.8)	-	16.9 (16.3, 17.6)
Cognition	15.2 (14.6, 15.9)	7.8 (7.5, 8.1)	19.7 (19.0, 20.4)	-

ORs corresponding to the two functional domains (communication and self-care) in Mauritius, Morocco, and Senegal:

**Predictors →<br><br>Response var ↓**	**Communication**	**Self-care**
Mauritius		
Hearing	120.6 (109.9, 132.3)	19.6 (17.9, 21.6)
Seeing	18.5 (16.8, 20.4)	14.7 (13.6, 15.9)
Mobility	72.5 (66.7, 78.9)	70.6 (65.8, 75.7)
Cognition	157.0 (146.0, 168.8)	88.2 (82.1, 94.6)

Self-care	138.0 (126.0, 151.1)	-
Communication	-	151.3 (138.9, 164.8)
Morocco		
Hearing	66.8 (66.1, 67.5)	16.1 (16.0, 16.3)
Seeing	16.1 (16.0, 16.3)	11.6 (11.5, 11.7)
Mobility	45.9 (45.4, 46.4)	91.6 (90.8, 92.4)
Cognition	297.5 (294.5, 300.6)	144.5 (143.3, 145.8)
Self-care	354.8 (350.7, 359.0)	-
Communication	-	301.4 (298.2, 304.7)
Senegal		
Hearing	195.7 (188.9, 202.6	54.9 (53.1, 56.7)
Seeing	20.6 (19.8, 21.3)	24.7 (23.9, 25.4)
Mobility	55.1 (53.2, 57.0)	201.6 (195.4, 208.0)
Cognition	314.9 (304.4, 325.7)	238.1 (231.1, 245.3)
Self-care	252.5 (243.7, 261.5)	-
Communication	-	259.1 (250.5, 268.0)

- Second threshold: A functional difficulty in a given domain was assigned 0 if people responded with "no difficulty" or "some difficulty" or "a lot of difficulty" and 1 if people responded with "unable to do" to the disability questions asked.

Table S4: *Sensitivity analysis (adjusted estimates):* Overall odds ratios in the presence of all control variables, along with their 95% CIs. All the p-values were <0.001 corresponding to each odd ratio implying statistically significant results at the 5% threshold. 988060, 23983300, 7288742, 35894738, 64267057, 2478630, were the cell counts used in the computation of ORs for Mauritius, Morocco, Senegal, Myanmar, Vietnam, and Uruguay respectively.

ORs corresponding to the four core functional domains among all countries included (n = 6):

Predictors → <br>Response var ↓	Hearing	Seeing	Mobility	Cognition
Mauritius				
Hearing	-	36.0 (29.4, 44.1)	19.6 (16.6, 23.2)	41.2 (34.6, 49.2)
Seeing	28.5 (23.1, 35.3)	-	20.5 (17.7, 23.7)	22.5 (18.8, 27.0)
Mobility	16.3 (13.8, 19.4)	22.2 (19.3, 25.6)	-	103.6 (92.6, 115.8)
Cognition	46.5 (39.1, 55.2)	30.5 (25.6, 36.3)	121.1 (109.1, 134.4)	-
Morocco				
Hearing	-	103.7 (101.3, 106.2)	49.6 (48.5, 50.7)	99.3 (97.2, 101.5)
Seeing	94.5 (92.3, 96.7)	-	30.6 (30.1, 31.2)	44.7 (43.7, 45.7)
Mobility	45.5 (44.5, 46.5)	30.9 (30.3, 31.4)	-	114.4 (112.4, 116.4)
Cognition	102.5 (100.3, 104.7)	50.2 (49.1, 51.3)	116.6 (114.7, 118.6)	-
Senegal				
Hearing	-	330.1 (310.9, 350.4)	469.3 (439.6, 500.9)	1137.0 (1068.3, 1210.3)
Seeing	325.9 (306.6 346.4)	-	93.0 (88.3, 98.0)	136.7 (128.6, 145.3)
Mobility	469.2 (438.0 502.5)	88.8 (84.3, 93.5)	-	510.9 (480.2, 543.6)
Cognition	1126.6 (1058.3, 1199.3)	143.6 (135.2, 152.5)	524.4 (494.2, 556.5)	-

Myanmar				
Hearing	-	107.8 (104.5, 111.2)	68.5 (66.5, 70.6)	224.9 (219.2, 230.7)
Seeing	90.6 (87.7, 93.6)	-	42.5 (41.5, 43.6)	50.6 (49.2, 52.0)
Mobility	59.5 (57.8, 61.4)	45.7 (44.6, 46.8)	-	213.5 (208.9, 218.1)
Cognition	226.6 (221.0, 232.4)	66.0 (64.2, 67.8)	238.6 (233.7, 243.6)	-
Vietnam				
Hearing	-	293.9 (288.5 299.3)	172.1 (169.3, 175.0)	544.3 (536.2, 552.5)
Seeing	257.0 (252.2, 261.8)	-	122.0 (120.0, 124.1)	152.6 (150.0, 155.1)
Mobility	150.2 (147.6, 152.8)	117.8 (115.9, 119.8)	-	257.3 (253.7, 260.8)
Cognition	545.4 (537.3, 553.6)	176.9 (174.1, 179.8)	283.2 (279.4, 287.0)	-
Uruguay				
Hearing	-	31.2 (27.1, 35.9)	14.6 (13, 16.5)	57.8 (51.3, 65.1)
Seeing	27.0 (23.4, 31.2)	-	14.1 (12.8, 15.5)	19.7 (17.1, 22.6)
Mobility	12.3 (10.9, 13.9)	14.3 (13.1, 15.7)	-	81.6 (75.4, 88.4)
Cognition	62.4 (55.5, 70.1)	24.7 (21.6, 28.4)	94.7 (88.0, 102.0)	-

ORs corresponding to the two functional domains (communication and self-care) in Mauritius, Morocco, and Senegal:

Predictors → Response var ↓	Communication	Self-care
Mauritius		
Hearing	728.7 (635.2, 835.9)	35.3 (29.8, 41.8)
Seeing	21.6 (17.4, 26.9)	24.0 (20.6, 27.9)
Mobility	121.9 (107.5, 138.4)	141.1 (128.9, 154.4)
Cognition	235.2 (210.3, 263.0)	206.7 (184.9, 231.0)
Self-care	134.5 (117.1, 154.5)	-
Communication	-	143.5 (126.3, 163.1)
Morocco		
Hearing	401.5 (393.3, 409.9)	51.1 (50.0, 52.2)
Seeing	50.3 (49.1, 51.6)	32.8 (32.3, 33.4)
Mobility	140.5 (137.8, 143.2)	282.5 (278.8, 286.2)
Cognition	697.2 (685.0, 709.6)	528.5 (519.5, 537.6)
Self-care	683.4 (670.2, 696.9)	-
Communication	-	550.3 (540.4, 560.3)
Senegal		
Hearing	1967.6 (1849.2, 2093.5)	493.5 (462.0, 527.1)
Seeing	106.8 (100.2, 113.9)	101.8 (96.4, 107.5)
Mobility	390.2 (366.4, 415.5)	1379.2 (1307.6, 1454.6)
Cognition	1359.8 (1282.7, 1441.4)	1389.6 (1309.0, 1475.2)
Self-care	895.1 (841.2, 952.4)	-

Communication	-	868.5 (818.8, 921.1)

Part 2: Crude ORs estimates

Table S5: *Main analysis (crude estimates):* Odds ratios of having a difficulty in a given domain (e.g. hearing) given a difficulty in another domain (e.g. seeing), along with their 95% CIs. All the p-values were <0.001 corresponding to each odd ratio implying statistically significant results at the 5% threshold. 988060, 23983300, 7288742, 35894738, 64267057, 2478630, were the cell counts used in the computation of ORs for Mauritius, Morocco, Senegal, Myanmar, Vietnam, and Uruguay respectively.

**Predictors →**<br>**Response var ↓**	**Hearing**	**Seeing**	**Mobility**	**Cognition**
Mauritius				
Hearing	-	-	-	-
Seeing	71.2 (67.8, 74.9)	-	-	-
Mobility	45.3 (43.2, 47.6)	36.2 (35, 37.4)	-	-
Cognition	51.1 (48.4, 54)	28.7 (27.5, 30)	42.5 (40.8, 44.2)	-
Morocco				
Hearing	-	-	-	-
Seeing	36.8 (36.7, 37.0)	-	-	-
Mobility	32.5 (32.4, 32.6)	22.3 (22.2, 22.4)	-	-

Cognition	31.9 (31.7, 32)	16.8 (16.7, 16.8)	41.2 (41, 41.4)	-
Senegal				
Hearing	-	-	-	-
Seeing	40.2 (39.8, 40.7)	-	-	-
Mobility	46.5 (46, 47.1)	27.9 (27.7, 28.2)	-	-
Cognition	64.6 (63.8, 65.4)	26.4 (26.1, 26.7)	61.9 (61.1, 62.6)	-
Myanmar				
Hearing	-	-	-	-
Seeing	42.9 (42.7, 43.2)	-	-	-
Mobility	40.2 (40, 40.4)	33.6 (33.5, 33.8)	-	-
Cognition	61.4 (61, 61.7)	39.7 (39.5, 39.9)	94.3 (93.8, 94.8)	-
Vietnam				
Hearing	-	-	-	-
Seeing	65.1 (64.9, 65.3)	-	-	-
Mobility	73.6 (73.4, 73.8)	46.3 (46.2, 46.4)	-	-
Cognition	128.7 (128.3, 129.2)	55.3 (55.2, 55.5)	90.2 (89.9, 90.5)	-
Uruguay				

Hearing	-	-	-	-
Seeing	8 (7.9, 8.1)	-	-	-
Mobility	9.7 (9.5, 9.8)	7.4 (7.3, 7.4)	-	-
Cognition	8.2 (8, 8.3)	4.8 (4.7, 4.9)	9.6 (9.5, 9.8)	-

Table S6: *Main analysis (crude estimates):* Odds ratios of having a difficulty in a given domain (e.g. hearing) given a difficulty in another domain (communication or self-care), along with their 95% CIs. Only Mauritius, Morocco, and Senegal, have data available for communication and self-care domains. All the p-values were <0.001 corresponding to each odd ratio implying statistically significant results at the 5% threshold. 988060, 23983300, 7288742, 35894738, 64267057, 2478630, were the cell counts used in the computation of ORs for Mauritius, Morocco, Senegal, Myanmar, Vietnam, and Uruguay respectively.

**Predictors** → **Response var ↓**	**Communication**	**Self-care**
Mauritius		
Hearing	92.9 (87.5, 98.6)	37.9 (36, 39.8)
Seeing	26.3 (24.9, 27.8)	27.5 (26.5, 28.5)
Mobility	66.7 (63.3, 70.3)	49 (47.5, 50.6)
Cognition	146.2 (138.5, 154.4)	80.2 (77, 83.5)
Self-care	108 (102.3, 114)	-
Communication	-	108 (102.3, 114)
Morocco		

Hearing	36.1 (35.8, 36.3)	31.1 (31, 31.3)
Seeing	12.6 (12.5, 12.7)	18.0 (17.9, 18.0)
Mobility	34.6 (34.4, 34.8)	98.7 (98.1, 99.4)
Cognition	207.4 (205.8, 209.1)	127.7 (126.9, 128.4)
Self-care	230.2 (228.4, 232.1)	-
Communication	-	230.2 (228.4, 232.1)
Senegal		
Hearing	48.4 (47.6, 49.1)	55.5 (54.7, 56.3)
Seeing	13.1 (12.9, 13.3)	24.1 (23.8, 24.4)
Mobility	27.2 (26.8, 27.6)	101.6 (100, 103.1)
Cognition	89.5 (88.1, 90.9)	145.1 (143, 147.2)
Self-care	119.4 (117.4, 121.3)	-
Communication	-	119.4 (117.4, 121.3)

Age-specific ORs estimates (crude):

Table S7: *Main analysis (crude estimates):* Age-specific odds ratios in the presence of all control variables, along with their 95% CIs. All the p-values were <0.001 corresponding to each odd ratio implying statistically significant results at the 5% threshold.

1. 45+ age-group:

ORs corresponding to the four core functional domains among all countries included (n = 6):

**Predictors →**<br>**Response var ↓**	**Hearing**	**Seeing**	**Mobility**	**Cognition**
Mauritius				
Hearing	-	-	-	-
Seeing	38.1 (36.1, 40.2)	-	-	-
Mobility	25.2 (24, 26.6)	20 (19.3, 20.8)	-	-
Cognition	34.3 (32.4, 36.4)	20.2 (19.2, 21.2)	30.5 (29, 31.9)	-
Morocco				
Hearing	-	-	-	-
Seeing	18.8 (18.7, 18.9)	-	-	-
Mobility	14.7 (14.7, 14.8)	11.2 (11.2, 11.3)	-	-
Cognition	18.1 (18, 18.2)	11.3 (11.2, 11.4)	26.6 (26.5, 26.8)	-
Senegal				
Hearing	-	-	-	-
Seeing	18.8 (18.5, 19.1)	-	-	-
Mobility	21.0 (20.7, 21.2)	12.2 (12.1, 12.4)	-	-

Cognition	27.9 (27.5, 28.3)	12.3 (12.2, 12.5)	30.8 (30.4, 31.3)	-
Myanmar				
Hearing	-	-	-	-
Seeing	21.5 (21.4, 21.6)	-	-	-
Mobility	20.2 (20.1, 20.3)	16.5 (16.4, 16.6)	-	-
Cognition	32.8 (32.6, 33)	23.6 (23.4, 23.7)	57.6 (57.2, 58)	-
Vietnam				
Hearing	-	-	-	-
Seeing	26.9 (26.8, 27.0)	-	-	-
Mobility	29.7 (29.6, 29.8)	19.0 (18.9, 19.1)	-	-
Cognition	55.1 (54.9, 55.3)	27.4 (27.3, 27.4)	42.2 (42.1, 42.4)	-
Uruguay				
Hearing	-	-	-	-
Seeing	4.8 (4.7, 4.8)	-	-	-
Mobility	5.2 (5.1, 5.3)	4.3 (4.2, 4.3)	-	-
Cognition	6.7 (6.6, 6.8)	4.5 (4.4, 4.6)	9.0 (8.8, 9.2)	-

ORs corresponding to the two functional domains (communication and self-care) in Mauritius, Morocco, and Senegal.

Predictors →<br><br>Response var ↓	Communication	Self-care
Mauritius		
Hearing	52.4 (48.9, 56.1)	26.5 (25.1, 28)
Seeing	17.1 (16.1, 18.3)	17.4 (16.7, 18.1)
Mobility	49.1 (45.9, 52.5)	32.5 (31.2, 33.8)
Cognition	75 (70.3, 80.1)	45.3 (43.1, 47.6)
Self-care	71.9 (67.4, 76.8)	-
Communication	-	71.9 (67.4, 76.8)
Morocco		
Hearing	23.5 (23.3, 23.7)	15.8 (15.7, 15.9
Seeing	9.9 (9.8, 10.0)	10.6 (10.5, 10.7)
Mobility	26.5 (26.2, 26.7)	63.1 (62.5, 63.6)
Cognition	118.5 (117.3, 119.8)	56.4 (56, 56.8)
Self-care	145.4 (143.8, 147.1)	-
Communication	-	145.4 (143.8, 147.1)
Senegal		
Hearing	26.8 (26.3, 27.4)	23.8 (23.4, 24.2)

Seeing	7.9 (7.8, 8.1)	10.8 (10.6, 11.0)
Mobility	19.7 (19.3, 20.1)	52.2 (51.1, 53.3)
Cognition	53.7 (52.5, 54.9)	58.8 (57.7, 59.8)
Self-care	70.8 (69.3, 72.4)	-
Communication	-	70.8 (69.3, 72.4)

2. '18-44' age-group

ORs corresponding to the four core functional domains among all countries included (n = 6):

**Predictors →**<br>**Response var ↓**	**Hearing**	**Seeing**	**Mobility**	**Cognition**
Mauritius				
Hearing	-	-	-	-
Seeing	116.7 (98.1, 138.9)	-	-	-
Mobility	40.3 (33, 49.2)	36.2 (31.7, 41.4)	-	-
Cognition	60.9 (51.1, 72.4)	34.7 (30.5, 39.5)	65.4 (59.5, 72)	-
Morocco				

Hearing	-	-	-	-
Seeing	25.9 (25.6, 26.2)	-	-	-
Mobility	33.4 (33, 33.8)	15.3 (15.1, 15.4)	-	-
Cognition	41.4 (40.9, 41.9)	12.5 (12.4, 12.7)	47.6 (47.1, 48.1)	-
Senegal				
Hearing	-	-	-	-
Seeing	34.4 (33.6, 35.3)	-	-	-
Mobility	43.2 (42.1, 44.2)	22 (21.5, 22.4)	-	-
Cognition	73.2 (71.3, 75.2)	20.5 (19.9, 21)	52.5 (51.3, 53.7)	-
Myanmar				
Hearing	-	-	-	-
Seeing	33.9 (33.3, 34.5)	-	-	-
Mobility	33.7 (33.1, 34.4)	29.4 (28.9, 29.9)	-	-
Cognition	70.8 (69.7, 72)	30.6 (30.2, 31.1)	78.9 (77.9, 80)	-
Vietnam				
Hearing	-	-	-	-
Seeing	73.8 (73.2, 74.5)	-	-	-

Mobility	98.1 (97.2, 99)	48.1 (47.7, 48.5)	-	-
Cognition	214.5 (212.6, 216.4)	46.8 (46.5, 47.2)	93.8 (93.1, 94.6)	-
Uruguay				
Hearing	-	-	-	-
Seeing	6.3 (6, 6.6)	-	-	-
Mobility	9.7 (9.2, 10.3)	5.6 (5.4, 5.8)	-	-
Cognition	13 (12.4, 13.6)	4.5 (4.4, 4.7)	17.2 (16.5, 17.9)	-

ORs corresponding to the two functional domains (communication and self-care) in Mauritius, Morocco, and Senegal:

**Predictors →** **Response var ↓**	**Communication**	**Self-care**
Mauritius		
Hearing	386.2 (329.6, 452.5)	86.6 (74.3, 100.9)
Seeing	48.8 (42.1, 56.6)	138.8 (125.7, 153.3)
Mobility	118.6 (106.4, 132.2)	355.1 (321.8, 391.9)
Cognition	388.9 (349.6, 432.6)	191.3 (176.4, 207.3)
Self-care	168.9 (151.8, 188)	-
Communication	-	168.9 (151.8, 188)

Morocco		
Hearing	79.3 (78.4, 80.3)	44.3 (43.7, 44.8)
Seeing	12.3 (12.1, 12.4)	14.2 (14.1, 14.4)
Mobility	54.4 (53.8, 55)	128 (126.5, 129.5)
Cognition	381.9 (376.9, 386.9)	313.2 (309.2, 317.2)
Self-care	483 (476.4, 489.6)	-
Communication	-	483 (476.4, 489.6)
Senegal		
Hearing	61.2 (59.4, 63)	62.5 (60.6, 64.5)
Seeing	10.6 (10.2, 10.9)	19.7 (19.1, 20.4)
Mobility	23.4 (22.8, 24.1)	101.5 (98.8, 104.3)
Cognition	114.5 (111.5, 117.6)	212.6 (206.5, 218.8)
Self-care	149.1 (144.7, 153.7	-
Communication	-	149.1 (144.7, 153.7)

Sensitivity analyses (crude estimates):

Country-specific estimates:

- First threshold: A functional difficulty in a given domain was assigned 0 if people responded with either "no difficulty" or "some difficulty" and 1 if people responded with either "a lot of difficulty" or "unable to do" to the disability questions asked.

Table S8: *Sensitivity analysis (crude estimates):* Overall odds ratios in the presence of all control variables, along with their 95% CIs. All the p-values were <0.001 corresponding to each odd ratio implying statistically significant results at the 5% threshold. 988060, 23983300, 7288742, 35894738, 64267057, 2478630, were the cell counts used in the computation of ORs for Mauritius, Morocco, Senegal, Myanmar, Vietnam, and Uruguay respectively.

ORs corresponding to the four core functional domains among all countries included (n = 6):

**Predictors →<br><br>Response var ↓**	**Hearing**	**Seeing**	**Mobility**	**Cognition**
Mauritius				
Hearing	-	-	-	-
Seeing	82.2 (75.9, 89)	-	-	-
Mobility	53.6 (49.9, 57.6)	52.8 (50, 55.8)	-	-
Cognition	71.1 (65.7, 77)	37.3 (34.8, 40.1)	51.8 (49.1, 54.6)	-
Morocco				
Hearing	-	-	-	-
Seeing	52.9 (52.5, 53.2)	-	-	-
Mobility	34.1 (33.9, 34.4)	27.3 (27.1, 27.4)	-	-
Cognition	42.5 (42.2, 42.9)	22.3 (22.1, 22.5)	36.3 (36.1, 36.6)	-
Senegal				

Hearing	-	-	-	-
Seeing	102.2 (99.6, 104.8)	-	-	-
Mobility	102.5 (100, 105.1)	54.8 (53.8, 55.9)	-	-
Cognition	211.9 (206.1, 217.9)	58.7 (57.2, 60.2)	135.9 (132.7, 139.2)	-
Myanmar				
Hearing	-	-	-	-
Seeing	139.7 (137.8, 141.6)	-	-	-
Mobility	86.9 (85.7, 88)	96.8 (95.7, 97.9)	-	-
Cognition	169.8 (167.5, 172)	92.4 (91.2, 93.5)	209.3 (207, 211.7)	-
Vietnam				
Hearing	-	-	-	-
Seeing	272.9 (271, 274.8)	-	-	-
Mobility	185.8 (184.6, 187)	198.4 (197.1, 199.7)	-	-
Cognition	354.6 (352.2, 357.1)	186.4 (185.2, 187.6)	220.6 (219.2, 221.9)	-
Uruguay				
Hearing	-	-	-	-
Seeing	15.3 (14.8, 15.8)	-	-	-
Mobility	16.2 (15.8, 16.7)	13.5 (13.2, 13.8)	-	-

Cognition	17.7 (16.9, 18.4)	8.8 (8.5, 9.1)	16.9 (16.4, 17.4)	-

ORs corresponding to the two functional domains (communication and self-care) in Mauritius, Morocco, and Senegal:

**Predictors →<br><br>Response var ↓**	**Communication**	**Self-care**
Mauritius		
Hearing	209.8 (193.6, 227.4)	84.3 (77.8, 91.4)
Seeing	43.1 (39.6, 46.9)	65.8 (61.5, 70.4)
Mobility	80.6 (75.5, 86)	196.6 (185.4, 208.5)
Cognition	201.5 (188.1, 215.9)	127 (119.5, 135.1)
Self-care	174.5 (162.6, 187.4)	-
Communication	-	174.5 (162.6, 187.4)
Morocco		
Hearing	66.9 (66.4, 67.5)	41.1 (40.8, 41.4)
Seeing	21.5 (21.3, 21.7)	28.8 (28.6, 29)
Mobility	37.8 (37.5, 38.1)	118.7 (117.8, 119.5)

Cognition	305 (302.1, 308)	166.5 (165.2, 167.8)
Self-care	230.2 (228, 232.3)	-
Communication	-	230.2 (228, 232.3)
Senegal		
Hearing	248.9 (241.5, 256.5)	144.9 (140.7, 149.1)
Seeing	36.9 (35.7, 38.1)	67.8 (66.1, 69.5)
Mobility	69.9 (67.9, 71.8)	318.9 (310.8, 327.3)
Cognition	350.8 (340.6, 361.2)	433.1 (421.4, 445.1)
Self-care	288.3 (279.9, 296.9)	-
Communication	-	288.3 (279.9, 296.9)

- Second threshold: A functional difficulty in a given domain was assigned 0 if people responded with "no difficulty" or "some difficulty" or "a lot of difficulty" and 1 if people responded with "unable to do" to the disability questions asked.

Table S9: *Sensitivity analysis (crude estimates):* Overall odds ratios in the presence of all control variables, along with their 95% CIs. All the p-values were <0.001 corresponding to each odd ratio implying statistically significant results at the 5% threshold. 988060, 23983300, 7288742, 35894738, 64267057, 2478630, were the cell counts used in the computation of ORs for Mauritius, Morocco, Senegal, Myanmar, Vietnam, and Uruguay respectively.

ORs corresponding to the four core functional domains among all countries included (n = 6):

**Predictors →**<br><br>**Response var ↓**	**Hearing**	**Seeing**	**Mobility**	**Cognition**
Mauritius				
Hearing	-	-	-	-
Seeing	100.5 (83, 121.6)	-	-	-
Mobility	55.3 (47.7, 64.1)	74.2 (65.4, 84.1)	-	-
Cognition	87.8 (74.7, 103.1)	61.7 (52.3, 72.9)	149 (136.3, 162.8)	-
Morocco				
Hearing	-	-	-	-
Seeing	181.7 (178, 185.5)	-	-	-
Mobility	81.1 (79.5, 82.7)	84.9 (83.6, 86.4)	-	-
Cognition	130.3 (127.6, 133)	70.2 (68.8, 71.7)	117.1 (115.4, 118.9)	-
Senegal				
Hearing	-	-	-	-
Seeing	357.7 (338.1,378.4)	-	-	-
Mobility	465.6 (439.1, 493.7)	169.4 (161.5, 177.6)	-	-
Cognition	1245.6 (1171.9, 1323.8)	184.6 (174.5, 195.4)	570.3 (540.6, 601.6)	-

Myanmar				
Hearing	-	-	-	-
Seeing	247.6 (240.9, 254.4)	-	-	-
Mobility	147.9 (144.1, 151.9)	187.1 (183, 191.2)	-	-
Cognition	345 (336.9, 353.3)	141.5 (138.1, 145)	333.1 (327.1, 339.2)	-
Vietnam				
Hearing	-	-	-	-
Seeing	588.6 (579.3, 598)	-	-	-
Mobility	305.6 (301.4, 310)	400.5 (394.8, 406.3)	-	-
Cognition	761.6 (750.9, 772.5)	334.5 (329.7, 339.5)	375.5 (371.2, 379.8)	-
Uruguay				
Hearing	-	-	-	-
Seeing	63.5 (55.4, 72.8)	-	-	-
Mobility	30.4 (27.2, 34)	42.5 (38.9, 46.4)	-	-
Cognition	80.5 (71.8, 90.2)	36.3 (31.8, 41.5)	98.1 (92, 104.7)	-

ORs corresponding to the two functional domains (communication and self-care) in Mauritius, Morocco, and Senegal:

**Predictors →**	**Communication**	**Self-care**

Response var ↓		
Mauritius		
Hearing	897.9 (789.7, 1021.1)	96.8 (83.9, 111.6)
Seeing	49 (40.1, 59.9)	97.6 (85.6, 111.3)
Mobility	128.9 (116.6, 142.6)	417.1 (385.7, 451.1)
Cognition	285.6 (256.7, 317.8)	235.4 (214.6, 258.2)
Self-care	150.7 (135.4, 167.8)	-
Communication	-	150.7 (135.4, 167.8)
Morocco		
Hearing	403.3 (395.5, 411.3)	79.6 (78.1, 81.1)
Seeing	60.9 (59.6 62.3)	82.2 (80.8, 83.5)
Mobility	107.7 (106, 109.4)	434.9 (429.5, 440.3)
Cognition	700.4 (688.5, 712.6)	429.5 (423, 436.1)
Self-care	369.1 (363.3, 375)	-
Communication	-	369.1 (363.3, 375)
Senegal		
Hearing	2047.1 (1926.1, 2175.8)	512 (482.1, 543.7)

Seeing	128 (120.6, 135.9)	184.7 (175.7, 194.1)
Mobility	380.7 (360.6, 401.9)	2055.5 (1956.1, 2160)
Cognition	1443.2 (1363.5, 1527.6)	1342.8 (1271.8, 1417.6)
Self-care	757.9 (718.6, 799.4	-
Communication	-	757.9 (718.6, 799.4)
